# Aqua­[1,3-bis­(benzimidazol-2-yl)-2-oxapropane]­diethano­lmanganese(II) dipicrate ethanol disolvate

**DOI:** 10.1107/S1600536810030357

**Published:** 2010-08-04

**Authors:** Tao Sun, Ke Li, Yulin Lai, Ruihuan Chen, Huilu Wu

**Affiliations:** a School of Chemical and Biological Engineering, Lanzhou Jiaotong University, Lanzhou 730070, People’s Republic of China

## Abstract

In the title complex, [Mn(C_16_H_14_N_4_O)(C_2_H_5_OH)_2_(H_2_O)](C_6_H_2_N_3_O_7_)_2_·2C_2_H_5_OH, the Mn^II^ ion is in a distorted octa­hedral coordination environment, defined by an MnN_2_O_4_ donor set. The 1,3-bis­(benz­imid­azol-2-yl)-2-oxapropane ligand is tridentate. In the crystal structure, inter­molecular N—H⋯O and O—H⋯O hydrogen bonds link the components into a three-dimensional network. The O atoms of one of the nitro groups are disordered over two sets of sites with refined occupancies of 0.577 (11) and 0.423 (11).

## Related literature

For the applications of benzimidazole and bis-benzimidazole compounds, see: Chang *et al.* (2008 ▶); Harrell *et al.* (2004 ▶); Holland & Tolman (2000 ▶). 
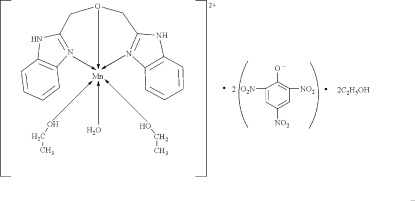


## Experimental

### 

#### Crystal data

[Mn(C_16_H_14_N_4_O)(C_2_H_6_O)_2_(H_2_O)](C_6_H_2_N_3_O_7_)_2_·2C_2_H_6_O
*M* *_r_* = 991.75
Monoclinic, 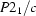

*a* = 10.0519 (3) Å
*b* = 24.8584 (8) Å
*c* = 18.0291 (7) Å
β = 98.504 (1)°
*V* = 4455.5 (3) Å^3^
*Z* = 4
Mo *K*α radiation
μ = 0.39 mm^−1^
*T* = 153 K
0.39 × 0.25 × 0.22 mm

#### Data collection

Bruker SMART APEXII diffractometer
Absorption correction: multi-scan (*SADABS*; Sheldrick, 1996 ▶) *T*_min_ = 0.864, *T*_max_ = 0.920
32660 measured reflections
7806 independent reflections
4707 reflections with *I* > 2σ(*I*)
*R*_int_ = 0.045

#### Refinement

*R*[*F* ^2^ > 2σ(*F* ^2^)] = 0.074
*wR*(*F* ^2^) = 0.255
*S* = 1.07
7806 reflections
603 parameters
8 restraints
H-atom parameters constrained
Δρ_max_ = 0.73 e Å^−3^
Δρ_min_ = −0.97 e Å^−3^

### 

Data collection: *APEX2* (Bruker, 2000 ▶); cell refinement: *SAINT* (Bruker, 2000 ▶); data reduction: *SAINT*; program(s) used to solve structure: *SHELXS97* (Sheldrick, 2008 ▶); program(s) used to refine structure: *SHELXL97* (Sheldrick, 2008 ▶); molecular graphics: *SHELXTL* (Sheldrick, 2008 ▶) and *PLATON* (Spek, 2009 ▶); software used to prepare material for publication: *SHELXL97*.

## Supplementary Material

Crystal structure: contains datablocks global, I. DOI: 10.1107/S1600536810030357/lh5083sup1.cif

Structure factors: contains datablocks I. DOI: 10.1107/S1600536810030357/lh5083Isup2.hkl

Additional supplementary materials:  crystallographic information; 3D view; checkCIF report

## Figures and Tables

**Table 1:** Hydrogen-bond geometry (Å, °)

*D*—H⋯*A*	*D*—H	H⋯*A*	*D*⋯*A*	*D*—H⋯*A*
O1—H1*W*⋯O5^i^	0.93	1.99	2.758 (4)	139
O1—H1*W*⋯O6^i^	0.93	2.07	2.854 (4)	141
O1—H2*W*⋯O5	0.93	1.99	2.758 (4)	139
O1—H2*W*⋯O11	0.93	2.11	2.898 (4)	141
O2—H2⋯O20^ii^	0.95	1.76	2.659 (7)	157
O3—H3⋯O17^iii^	0.95	2.09	2.864 (4)	138
O3—H3⋯O18^iii^	0.95	2.37	3.289 (5)	163
N2—H2*B*⋯O12^i^	0.88	1.92	2.676 (4)	142
N2—H2*B*⋯O18^i^	0.88	2.25	2.973 (5)	140
N4—H4*B*⋯O15^iv^	0.88	2.10	2.907 (5)	152
N4—H4*B*⋯O16^iv^	0.88	2.38	3.161 (5)	148
O19—H19⋯O8	0.93	2.35	3.278 (13)	179
O20—H20⋯O19	0.93	1.88	2.807 (13)	179

Symmetry codes: (i) 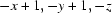; (ii) 
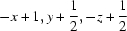
; (iii) 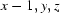; (iv) 
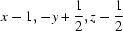
.

**Fig. 1.**
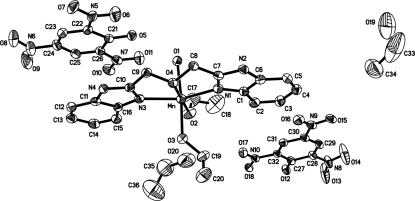
The asymmetric unit of the title compound showing 30% ellipsoids.

**Fig. 2.**
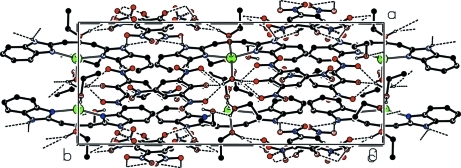
Part of the crystal structure of the title compound showing hydrogen bonds as dashed lines. Only the H atoms involved in hydrogen bonds are shown.

## supplementary crystallographic information

### Comment

Bis-benzimidazoles are known to be strong chelating agents coordinating through both of the C=N  group nitrogen atoms. In addition, as a typical multidentate ligand they have polymer-forming characteristics. In bioinorganic chemistry, they have been used extensively to help model the active sites of metalloproteins (Holland  & Tolman,  2000). The benzimidazole ring system is present in clinically approved anthelmintics, antiulcers, antivirals, and antihistamines (Harrell, *et al.*,  2004). Recently, there have been reports on benzimidazole  derivatives exhibiting antitumor and antimicrobial properties and acting as thrombopoietin receptor agonists (Chang, *et al.*, 2008).

We have attempted to prepare organic-inorganic hybrid materials with manganese salts using 1,3-bis(benzimidazol-2-yl)-2-oxapropane (OBB). Herein, we report the crystal structure of the title compound. The asymmetric unit of the title compound is shown in Fig. 1. The Mn^II^ ion is coordinated in a distorted octahedral coordination environment. The 1,3-bis(benzimidazol-2-yl)-2-oxapropane ligand acts as tridentate. In the crystal structure, intermolecular N—H···O and O—H···O hydrogen bonds link the components of the structure into a three-dimensional network (Fig. 2).

### Experimental

To a stirred solution of 1,3-bis(benzimidazol-2-yl)-2-oxapropane (91.6 mg, 0.2 mmol) in hot ethanol (10 ml), Mn(C_6_H_2_N_3_O_7_)_2_ (102.9 mg, 0.2 mmol) in ethanol (5 ml) was added. A yellow crystalline product formed rapidly. The precipitate was filtered off, washed with ethanol and Et_2_O, and dried *in vacuo*. The dried precipitate was dissolved in DMF to form a yellow solution into which diethylether was allowed to diffuse at room temperature. The yellow crystals suitable for X-ray diffraction studies were obtained after one week (Yield, 72%).

### Refinement

All H atoms were placed in calculated positions and refined in a riding-model approximation with; C—H = 0.95-0.99 Å, N-H = 0.88Å, O-H = 0.93-0.95Å and *U*_iso_(H) = 1.2 *U*_eq_(C, N) or *U*_iso_(H) = 1.5 *U*_eq_(C_methyl_, O).

### Crystal data

**Table:**

[Mn(C_16_H_14_N_4_O)(C_2_H_6_O)_2_(H_2_O)](C_6_H_2_N_3_O_7_)_2_·2C_2_H_6_O	*F*(000) = 2060
*M**_r_* = 991.75	*D*_x_ = 1.478 Mg m^−^^3^
Monoclinic, *P*2_1_/*c*	Mo *K*α radiation, λ = 0.71073 Å
Hall symbol: -P 2ybc	Cell parameters from 8263 reflections
*a* = 10.0519 (3) Å	θ = 3.2–25.5°
*b* = 24.8584 (8) Å	µ = 0.39 mm^−^^1^
*c* = 18.0291 (7) Å	*T* = 153 K
β = 98.504 (1)°	Block, yellow
*V* = 4455.5 (3) Å^3^	0.39 × 0.25 × 0.22 mm
*Z* = 4	

### Data collection

**Table:**

Bruker SMART APEXII diffractometer	7806 independent reflections
Radiation source: fine-focus sealed tube	4707 reflections with *I* > 2σ(*I*)
graphite	*R*_int_ = 0.045
ω scans	θ_max_ = 25.0°, θ_min_ = 3.2°
Absorption correction: multi-scan (*SADABS*; Sheldrick, 1996)	*h* = −11→11
*T*_min_ = 0.864, *T*_max_ = 0.920	*k* = −26→29
32660 measured reflections	*l* = −21→21

### Refinement

**Table:**

Refinement on *F*^2^	Primary atom site location: structure-invariant direct methods
Least-squares matrix: full	Secondary atom site location: difference Fourier map
*R*[*F*^2^ > 2σ(*F*^2^)] = 0.074	Hydrogen site location: inferred from neighbouring sites
*wR*(*F*^2^) = 0.255	H-atom parameters constrained
*S* = 1.07	*w* = 1/[σ^2^(*F*_o_^2^) + (0.1644*P*)^2^] where *P* = (*F*_o_^2^ + 2*F*_c_^2^)/3
7806 reflections	(Δ/σ)_max_ = 0.003
603 parameters	Δρ_max_ = 0.73 e Å^−^^3^
8 restraints	Δρ_min_ = −0.97 e Å^−^^3^

### Special details

**Table:**

Geometry. All esds (except the esd in the dihedral angle between two l.s. planes) are estimated using the full covariance matrix. The cell esds are taken into account individually in the estimation of esds in distances, angles and torsion angles; correlations between esds in cell parameters are only used when they are defined by crystal symmetry. An approximate (isotropic) treatment of cell esds is used for estimating esds involving l.s. planes.
Refinement. Refinement of F^2^ against ALL reflections. The weighted R-factor wR and goodness of fit S are based on F^2^, conventional R-factors R are based on F, with F set to zero for negative F^2^. The threshold expression of F^2^ > 2sigma(F^2^) is used only for calculating R-factors(gt) etc. and is not relevant to the choice of reflections for refinement. R-factors based on F^2^ are statistically about twice as large as those based on F, and R- factors based on ALL data will be even larger.

### Fractional atomic coordinates and isotropic or equivalent isotropic displacement parameters (Å^2^)

**Table:**

	*x*	*y*	*z*	*U*_iso_*/*U*_eq_	Occ. (<1)
Mn	0.28926 (6)	0.50170 (2)	0.15226 (4)	0.0402 (3)	
O1	0.4637 (3)	0.50371 (10)	0.09714 (18)	0.0451 (7)	
H1W	0.4967	0.5364	0.0826	0.068*	
H2W	0.5072	0.4715	0.0900	0.068*	
O2	0.3980 (4)	0.50978 (15)	0.2620 (2)	0.0687 (10)	
H2	0.3459	0.5085	0.3018	0.082*	
O3	0.1054 (4)	0.49324 (12)	0.2034 (2)	0.0597 (9)	
H3	0.0606	0.4596	0.1956	0.072*	
O4	0.1588 (3)	0.49524 (10)	0.03089 (16)	0.0380 (7)	
N1	0.2345 (3)	0.58284 (14)	0.10970 (19)	0.0407 (8)	
N2	0.1547 (3)	0.64007 (13)	0.01947 (19)	0.0401 (8)	
H2B	0.1184	0.6519	−0.0249	0.048*	
N3	0.2699 (3)	0.41580 (13)	0.11969 (19)	0.0387 (8)	
N4	0.2055 (3)	0.35199 (13)	0.03689 (19)	0.0406 (8)	
H4B	0.1703	0.3367	−0.0055	0.049*	
C1	0.2462 (4)	0.63501 (17)	0.1386 (2)	0.0410 (10)	
C2	0.2999 (4)	0.65352 (19)	0.2102 (3)	0.0485 (11)	
H2A	0.3319	0.6294	0.2497	0.058*	
C3	0.3040 (5)	0.70810 (19)	0.2202 (3)	0.0517 (12)	
H3A	0.3424	0.7220	0.2677	0.062*	
C4	0.2539 (5)	0.74418 (19)	0.1633 (3)	0.0516 (12)	
H4A	0.2583	0.7817	0.1733	0.062*	
C5	0.1986 (4)	0.72665 (17)	0.0935 (3)	0.0467 (11)	
H5A	0.1637	0.7510	0.0549	0.056*	
C6	0.1962 (4)	0.67122 (17)	0.0824 (2)	0.0419 (10)	
C7	0.1802 (4)	0.58830 (17)	0.0388 (2)	0.0393 (9)	
C8	0.1595 (4)	0.54227 (15)	−0.0147 (2)	0.0391 (10)	
H8A	0.0730	0.5460	−0.0485	0.047*	
H8B	0.2333	0.5405	−0.0454	0.047*	
C9	0.1768 (4)	0.44668 (16)	−0.0084 (2)	0.0408 (10)	
H9A	0.2485	0.4511	−0.0401	0.049*	
H9B	0.0924	0.4365	−0.0409	0.049*	
C10	0.2155 (4)	0.40456 (17)	0.0506 (2)	0.0387 (10)	
C11	0.2612 (4)	0.32574 (17)	0.1022 (2)	0.0414 (10)	
C12	0.2816 (4)	0.27105 (17)	0.1189 (3)	0.0480 (11)	
H12A	0.2526	0.2437	0.0833	0.058*	
C13	0.3455 (4)	0.2593 (2)	0.1892 (3)	0.0523 (12)	
H13A	0.3623	0.2228	0.2030	0.063*	
C14	0.3872 (5)	0.3002 (2)	0.2416 (3)	0.0539 (12)	
H14A	0.4323	0.2905	0.2898	0.065*	
C15	0.3647 (4)	0.35295 (18)	0.2253 (2)	0.0461 (11)	
H15A	0.3908	0.3800	0.2617	0.055*	
C16	0.3016 (4)	0.36626 (17)	0.1529 (2)	0.0392 (10)	
C17	0.5414 (7)	0.5170 (4)	0.2839 (4)	0.111 (3)	
H17A	0.5843	0.5187	0.2379	0.134*	
H17B	0.5775	0.4848	0.3125	0.134*	
C18	0.5828 (9)	0.5670 (4)	0.3312 (6)	0.158 (4)	
H18A	0.6809	0.5678	0.3445	0.237*	
H18B	0.5413	0.5659	0.3771	0.237*	
H18C	0.5527	0.5993	0.3024	0.237*	
C19	0.0450 (6)	0.5308 (2)	0.2460 (4)	0.0798 (18)	
H19A	0.0727	0.5674	0.2328	0.096*	
H19B	0.0794	0.5249	0.2998	0.096*	
C20	−0.1021 (6)	0.5284 (3)	0.2355 (4)	0.099 (2)	
H20A	−0.1365	0.5555	0.2674	0.148*	
H20B	−0.1307	0.4926	0.2493	0.148*	
H20C	−0.1375	0.5356	0.1828	0.148*	
O5	0.5161 (3)	0.41792 (12)	0.00890 (15)	0.0447 (7)	
O6	0.3795 (4)	0.40081 (15)	−0.1255 (2)	0.0732 (11)	
O7	0.3734 (4)	0.32152 (15)	−0.1712 (2)	0.0725 (11)	
O8	0.5357 (7)	0.16990 (18)	−0.0341 (4)	0.126 (2)	
O9	0.6222 (7)	0.17536 (18)	0.0825 (4)	0.136 (2)	
O10	0.7047 (3)	0.34095 (14)	0.1990 (2)	0.0673 (10)	
O11	0.6462 (4)	0.41649 (13)	0.1475 (2)	0.0643 (9)	
N5	0.4079 (4)	0.35240 (16)	−0.1193 (2)	0.0524 (10)	
N6	0.5727 (7)	0.1957 (2)	0.0240 (4)	0.102 (2)	
N7	0.6505 (4)	0.36722 (16)	0.1451 (2)	0.0509 (10)	
C21	0.5280 (4)	0.36791 (18)	0.0124 (2)	0.0419 (10)	
C22	0.4763 (5)	0.33195 (17)	−0.0490 (3)	0.0465 (11)	
C23	0.4907 (5)	0.2772 (2)	−0.0454 (3)	0.0614 (14)	
H23A	0.4560	0.2555	−0.0872	0.074*	
C24	0.5567 (5)	0.25352 (19)	0.0198 (3)	0.0629 (14)	
C25	0.6078 (5)	0.28421 (19)	0.0812 (3)	0.0576 (13)	
H25A	0.6533	0.2676	0.1252	0.069*	
C26	0.5921 (4)	0.33915 (17)	0.0778 (3)	0.0458 (11)	
N8	0.9544 (5)	0.17124 (16)	0.1081 (3)	0.0783 (15)	
N9	1.1077 (4)	0.21015 (19)	0.3675 (2)	0.0564 (11)	
O12	0.9230 (4)	0.28313 (12)	0.08169 (19)	0.0605 (9)	
O13	0.9718 (8)	0.1816 (3)	0.0434 (3)	0.075 (3)*	0.577 (11)
O14	0.9109 (8)	0.1275 (2)	0.1270 (4)	0.081 (3)*	0.577 (11)
O13A	0.8804 (10)	0.1802 (4)	0.0484 (5)	0.083 (4)*	0.423 (11)
O14A	0.9955 (13)	0.1244 (3)	0.1159 (7)	0.097 (4)*	0.423 (11)
O15	1.1017 (3)	0.16073 (15)	0.3786 (2)	0.0662 (10)	
O16	1.1480 (4)	0.24173 (17)	0.4180 (2)	0.0685 (10)	
O17	1.0423 (3)	0.38875 (13)	0.25535 (19)	0.0590 (9)	
O18	0.9626 (3)	0.38007 (12)	0.13932 (18)	0.0517 (8)	
C27	0.9682 (4)	0.26778 (18)	0.1456 (3)	0.0481 (11)	
C28	0.9854 (5)	0.21100 (18)	0.1660 (3)	0.0523 (12)	
C29	1.0283 (5)	0.19286 (19)	0.2356 (3)	0.0538 (12)	
H29A	1.0340	0.1553	0.2453	0.065*	
C30	1.0641 (4)	0.22935 (19)	0.2930 (3)	0.0472 (11)	
C31	1.0545 (4)	0.28391 (18)	0.2804 (3)	0.0459 (11)	
H31A	1.0793	0.3085	0.3204	0.055*	
C32	1.0087 (4)	0.30245 (17)	0.2095 (3)	0.0429 (10)	
N10	1.0031 (3)	0.36034 (15)	0.2009 (2)	0.0456 (9)	
O19	0.7129 (15)	0.0593 (3)	−0.0039 (7)	0.280 (6)	
H19	0.6622	0.0906	−0.0120	0.420*	
C33	0.8305 (13)	0.0560 (5)	−0.0371 (9)	0.292 (14)	
H33A	0.8446	0.0190	−0.0547	0.351*	
H33B	0.9103	0.0672	−0.0016	0.351*	
C34	0.8024 (13)	0.0960 (5)	−0.1043 (6)	0.169 (5)	
H34A	0.8783	0.0956	−0.1327	0.253*	
H34B	0.7909	0.1324	−0.0853	0.253*	
H34C	0.7202	0.0851	−0.1372	0.253*	
O20	0.7010 (7)	−0.0141 (3)	0.1132 (3)	0.141 (2)	
H20	0.7056	0.0099	0.0742	0.211*	
C35	0.6637 (14)	−0.0651 (4)	0.0749 (6)	0.173 (5)	
H35A	0.6185	−0.0878	0.1087	0.208*	
H35B	0.5970	−0.0573	0.0300	0.208*	
C36	0.7723 (13)	−0.0961 (6)	0.0511 (8)	0.214 (6)	
H36A	0.7349	−0.1271	0.0216	0.320*	
H36B	0.8329	−0.1086	0.0954	0.320*	
H36C	0.8224	−0.0735	0.0204	0.320*	

### Atomic displacement parameters (Å^2^)

**Table:**

	*U*^11^	*U*^22^	*U*^33^	*U*^12^	*U*^13^	*U*^23^
Mn	0.0467 (4)	0.0440 (4)	0.0288 (4)	0.0027 (3)	0.0021 (3)	0.0021 (3)
O1	0.0509 (17)	0.0410 (16)	0.0441 (19)	0.0022 (12)	0.0094 (14)	−0.0005 (12)
O2	0.069 (2)	0.097 (3)	0.037 (2)	0.0132 (19)	−0.0015 (17)	−0.0032 (18)
O3	0.072 (2)	0.0499 (19)	0.063 (2)	−0.0003 (15)	0.0297 (19)	−0.0059 (15)
O4	0.0453 (16)	0.0409 (16)	0.0264 (15)	−0.0014 (12)	0.0004 (12)	0.0018 (11)
N1	0.046 (2)	0.046 (2)	0.029 (2)	0.0010 (16)	0.0013 (15)	0.0035 (16)
N2	0.0444 (19)	0.0419 (19)	0.034 (2)	0.0032 (16)	0.0049 (15)	0.0081 (16)
N3	0.0424 (18)	0.0445 (19)	0.0288 (19)	0.0023 (16)	0.0039 (15)	0.0025 (15)
N4	0.049 (2)	0.0394 (19)	0.033 (2)	−0.0035 (16)	0.0043 (16)	−0.0026 (15)
C1	0.040 (2)	0.044 (2)	0.040 (3)	−0.0014 (19)	0.0064 (18)	0.0005 (19)
C2	0.053 (3)	0.055 (3)	0.037 (3)	0.001 (2)	0.005 (2)	−0.001 (2)
C3	0.052 (3)	0.055 (3)	0.048 (3)	−0.006 (2)	0.005 (2)	−0.012 (2)
C4	0.053 (3)	0.046 (3)	0.057 (3)	−0.005 (2)	0.013 (2)	−0.005 (2)
C5	0.050 (3)	0.041 (2)	0.050 (3)	−0.003 (2)	0.013 (2)	0.004 (2)
C6	0.039 (2)	0.047 (2)	0.040 (3)	−0.0027 (19)	0.0091 (19)	0.000 (2)
C7	0.040 (2)	0.044 (2)	0.035 (2)	−0.0012 (18)	0.0080 (18)	0.0044 (19)
C8	0.047 (2)	0.039 (2)	0.030 (2)	0.0014 (18)	0.0031 (18)	0.0055 (18)
C9	0.046 (2)	0.045 (2)	0.031 (2)	0.0011 (19)	0.0054 (18)	0.0008 (19)
C10	0.041 (2)	0.044 (2)	0.031 (2)	−0.0023 (18)	0.0054 (18)	−0.0025 (18)
C11	0.041 (2)	0.049 (3)	0.036 (2)	0.0030 (19)	0.0106 (19)	0.0059 (19)
C12	0.053 (3)	0.043 (2)	0.049 (3)	0.002 (2)	0.012 (2)	0.005 (2)
C13	0.054 (3)	0.051 (3)	0.055 (3)	0.006 (2)	0.016 (2)	0.013 (2)
C14	0.055 (3)	0.063 (3)	0.044 (3)	0.009 (2)	0.009 (2)	0.017 (2)
C15	0.052 (3)	0.050 (3)	0.035 (2)	0.004 (2)	0.004 (2)	0.005 (2)
C16	0.042 (2)	0.040 (2)	0.036 (2)	0.0058 (18)	0.0070 (18)	0.0012 (18)
C17	0.089 (5)	0.165 (7)	0.072 (5)	0.017 (5)	−0.015 (4)	−0.028 (5)
C18	0.110 (7)	0.207 (10)	0.143 (9)	0.032 (6)	−0.029 (6)	−0.047 (8)
C19	0.081 (4)	0.074 (4)	0.094 (5)	−0.004 (3)	0.046 (4)	−0.025 (3)
C20	0.076 (4)	0.118 (6)	0.102 (6)	0.016 (4)	0.014 (4)	−0.016 (4)
O5	0.0531 (18)	0.0412 (17)	0.0388 (18)	0.0014 (13)	0.0033 (14)	−0.0009 (13)
O6	0.090 (3)	0.060 (2)	0.060 (2)	−0.010 (2)	−0.0173 (19)	−0.0121 (19)
O7	0.074 (2)	0.077 (2)	0.066 (3)	−0.0082 (19)	0.0099 (19)	−0.038 (2)
O8	0.185 (6)	0.047 (3)	0.154 (6)	−0.004 (3)	0.049 (4)	−0.016 (3)
O9	0.215 (7)	0.048 (3)	0.142 (5)	0.018 (3)	0.022 (5)	0.026 (3)
O10	0.065 (2)	0.073 (2)	0.062 (2)	0.0082 (17)	0.0048 (18)	0.0288 (19)
O11	0.081 (2)	0.049 (2)	0.054 (2)	0.0006 (17)	−0.0159 (18)	0.0049 (16)
N5	0.053 (2)	0.052 (2)	0.054 (3)	−0.0099 (19)	0.0130 (19)	−0.015 (2)
N6	0.148 (6)	0.040 (3)	0.125 (6)	−0.006 (3)	0.047 (5)	−0.003 (3)
N7	0.048 (2)	0.055 (2)	0.050 (3)	0.0026 (19)	0.0088 (18)	0.015 (2)
C21	0.041 (2)	0.043 (3)	0.043 (3)	−0.0030 (19)	0.0125 (19)	−0.0008 (19)
C22	0.050 (3)	0.043 (3)	0.050 (3)	0.000 (2)	0.017 (2)	−0.006 (2)
C23	0.068 (3)	0.052 (3)	0.072 (4)	−0.011 (2)	0.033 (3)	−0.017 (3)
C24	0.071 (3)	0.039 (3)	0.086 (4)	0.002 (2)	0.033 (3)	0.007 (3)
C25	0.058 (3)	0.049 (3)	0.071 (4)	0.000 (2)	0.026 (3)	0.012 (3)
C26	0.044 (2)	0.043 (2)	0.054 (3)	−0.0011 (19)	0.016 (2)	0.003 (2)
N8	0.126 (4)	0.042 (2)	0.059 (3)	−0.009 (2)	−0.012 (3)	0.017 (2)
N9	0.047 (2)	0.070 (3)	0.053 (3)	0.011 (2)	0.010 (2)	0.027 (2)
O12	0.082 (2)	0.0488 (18)	0.046 (2)	−0.0060 (17)	−0.0055 (17)	0.0109 (15)
O15	0.060 (2)	0.078 (3)	0.062 (2)	0.0123 (18)	0.0136 (17)	0.0355 (19)
O16	0.074 (2)	0.087 (3)	0.043 (2)	0.017 (2)	0.0025 (18)	0.011 (2)
O17	0.078 (2)	0.0529 (19)	0.043 (2)	−0.0001 (16)	0.0018 (17)	0.0019 (16)
O18	0.0632 (19)	0.0477 (18)	0.0436 (19)	0.0062 (14)	0.0056 (15)	0.0143 (15)
C27	0.051 (3)	0.049 (3)	0.042 (3)	0.001 (2)	−0.001 (2)	0.014 (2)
C28	0.063 (3)	0.045 (3)	0.049 (3)	−0.007 (2)	0.006 (2)	0.007 (2)
C29	0.057 (3)	0.048 (3)	0.056 (3)	0.001 (2)	0.006 (2)	0.019 (2)
C30	0.045 (2)	0.055 (3)	0.041 (3)	0.004 (2)	0.007 (2)	0.015 (2)
C31	0.043 (2)	0.055 (3)	0.040 (3)	0.008 (2)	0.0060 (19)	0.010 (2)
C32	0.040 (2)	0.046 (2)	0.044 (3)	0.0045 (19)	0.0096 (19)	0.009 (2)
N10	0.044 (2)	0.050 (2)	0.043 (2)	0.0028 (17)	0.0068 (17)	0.0086 (19)
O19	0.45 (2)	0.109 (6)	0.316 (15)	−0.045 (9)	0.159 (14)	−0.037 (7)
C33	0.189 (13)	0.151 (13)	0.57 (4)	−0.059 (11)	0.17 (2)	−0.138 (18)
C34	0.245 (14)	0.125 (8)	0.140 (10)	−0.038 (8)	0.036 (9)	0.005 (7)
O20	0.195 (7)	0.160 (5)	0.072 (4)	−0.043 (4)	0.035 (4)	−0.030 (3)
C35	0.260 (15)	0.138 (9)	0.127 (9)	−0.058 (9)	0.047 (9)	−0.050 (7)
C36	0.172 (11)	0.272 (16)	0.184 (13)	0.080 (11)	−0.015 (9)	−0.025 (12)

### Geometric parameters (Å, °)

**Table:**

Mn—O2	2.123 (4)	C19—H19B	0.9900
Mn—O1	2.140 (3)	C20—H20A	0.9800
Mn—O3	2.194 (3)	C20—H20B	0.9800
Mn—N1	2.199 (3)	C20—H20C	0.9800
Mn—N3	2.215 (3)	O5—C21	1.250 (5)
Mn—O4	2.384 (3)	O6—N5	1.238 (5)
O1—H1W	0.9300	O7—N5	1.220 (5)
O1—H2W	0.9301	O8—N6	1.236 (8)
O2—C17	1.449 (8)	O9—N6	1.209 (8)
O2—H2	0.9500	O10—N7	1.229 (5)
O3—C19	1.404 (6)	O11—N7	1.227 (5)
O3—H3	0.9500	N5—C22	1.443 (6)
O4—C9	1.425 (5)	N6—C24	1.448 (7)
O4—C8	1.429 (5)	N7—C26	1.446 (6)
N1—C7	1.320 (5)	C21—C26	1.447 (6)
N1—C1	1.396 (5)	C21—C22	1.457 (6)
N2—C7	1.348 (5)	C22—C23	1.370 (6)
N2—C6	1.386 (5)	C23—C24	1.392 (8)
N2—H2B	0.8800	C23—H23A	0.9500
N3—C10	1.314 (5)	C24—C25	1.379 (7)
N3—C16	1.386 (5)	C25—C26	1.375 (6)
N4—C10	1.331 (5)	C25—H25A	0.9500
N4—C11	1.389 (5)	N8—O13	1.232 (5)
N4—H4B	0.8800	N8—O14A	1.236 (5)
C1—C6	1.393 (6)	N8—O13A	1.236 (5)
C1—C2	1.401 (6)	N8—O14	1.239 (5)
C2—C3	1.369 (6)	N8—C28	1.437 (6)
C2—H2A	0.9500	N9—O16	1.224 (5)
C3—C4	1.400 (7)	N9—O15	1.247 (5)
C3—H3A	0.9500	N9—C30	1.433 (6)
C4—C5	1.369 (6)	O12—C27	1.235 (5)
C4—H4A	0.9500	O17—N10	1.226 (5)
C5—C6	1.392 (6)	O18—N10	1.227 (4)
C5—H5A	0.9500	C27—C32	1.448 (6)
C7—C8	1.491 (6)	C27—C28	1.462 (6)
C8—H8A	0.9900	C28—C29	1.344 (6)
C8—H8B	0.9900	C29—C30	1.383 (7)
C9—C10	1.502 (6)	C29—H29A	0.9500
C9—H9A	0.9900	C30—C31	1.376 (6)
C9—H9B	0.9900	C31—C32	1.373 (6)
C11—C16	1.381 (6)	C31—H31A	0.9500
C11—C12	1.401 (6)	C32—N10	1.448 (6)
C12—C13	1.365 (7)	O19—C33	1.404 (9)
C12—H12A	0.9500	O19—H19	0.9299
C13—C14	1.409 (7)	C33—C34	1.560 (10)
C13—H13A	0.9500	C33—H33A	0.9900
C14—C15	1.356 (6)	C33—H33B	0.9900
C14—H14A	0.9500	C34—H34A	0.9800
C15—C16	1.403 (6)	C34—H34B	0.9800
C15—H15A	0.9500	C34—H34C	0.9800
C17—C18	1.531 (11)	O20—C35	1.466 (10)
C17—H17A	0.9900	O20—H20	0.9296
C17—H17B	0.9900	C35—C36	1.451 (14)
C18—H18A	0.9800	C35—H35A	0.9900
C18—H18B	0.9800	C35—H35B	0.9900
C18—H18C	0.9800	C36—H36A	0.9800
C19—C20	1.464 (8)	C36—H36B	0.9800
C19—H19A	0.9900	C36—H36C	0.9800
			
O2—Mn—O1	94.95 (14)	C17—C18—H18C	109.5
O2—Mn—O3	88.13 (15)	H18A—C18—H18C	109.5
O1—Mn—O3	175.05 (11)	H18B—C18—H18C	109.5
O2—Mn—N1	107.83 (13)	O3—C19—C20	114.3 (5)
O1—Mn—N1	89.80 (12)	O3—C19—H19A	108.7
O3—Mn—N1	92.95 (12)	C20—C19—H19A	108.7
O2—Mn—N3	110.56 (13)	O3—C19—H19B	108.7
O1—Mn—N3	86.88 (11)	C20—C19—H19B	108.7
O3—Mn—N3	88.41 (12)	H19A—C19—H19B	107.6
N1—Mn—N3	141.62 (13)	C19—C20—H20A	109.5
O2—Mn—O4	177.23 (12)	C19—C20—H20B	109.5
O1—Mn—O4	87.29 (11)	H20A—C20—H20B	109.5
O3—Mn—O4	89.76 (12)	C19—C20—H20C	109.5
N1—Mn—O4	70.50 (11)	H20A—C20—H20C	109.5
N3—Mn—O4	71.15 (10)	H20B—C20—H20C	109.5
Mn—O1—H1W	120.2	O7—N5—O6	120.5 (4)
Mn—O1—H2W	118.7	O7—N5—C22	119.8 (4)
H1W—O1—H2W	121.1	O6—N5—C22	119.7 (4)
C17—O2—Mn	128.3 (4)	O9—N6—O8	123.8 (6)
C17—O2—H2	115.8	O9—N6—C24	119.2 (7)
Mn—O2—H2	115.8	O8—N6—C24	117.0 (7)
C19—O3—Mn	128.7 (3)	O11—N7—O10	121.1 (4)
C19—O3—H3	115.6	O11—N7—C26	120.0 (4)
Mn—O3—H3	115.6	O10—N7—C26	118.9 (4)
C9—O4—C8	113.3 (3)	O5—C21—C26	124.1 (4)
C9—O4—Mn	114.9 (2)	O5—C21—C22	123.4 (4)
C8—O4—Mn	115.1 (2)	C26—C21—C22	112.5 (4)
C7—N1—C1	105.4 (3)	C23—C22—N5	115.1 (4)
C7—N1—Mn	118.6 (3)	C23—C22—C21	123.4 (5)
C1—N1—Mn	136.0 (3)	N5—C22—C21	121.4 (4)
C7—N2—C6	107.4 (4)	C22—C23—C24	119.5 (5)
C7—N2—H2B	126.3	C22—C23—H23A	120.2
C6—N2—H2B	126.3	C24—C23—H23A	120.2
C10—N3—C16	104.9 (3)	C25—C24—C23	121.2 (5)
C10—N3—Mn	117.7 (3)	C25—C24—N6	118.9 (6)
C16—N3—Mn	137.4 (3)	C23—C24—N6	119.9 (6)
C10—N4—C11	107.1 (3)	C26—C25—C24	119.2 (5)
C10—N4—H4B	126.4	C26—C25—H25A	120.4
C11—N4—H4B	126.4	C24—C25—H25A	120.4
C6—C1—N1	109.1 (4)	C25—C26—N7	114.4 (4)
C6—C1—C2	120.4 (4)	C25—C26—C21	124.1 (5)
N1—C1—C2	130.4 (4)	N7—C26—C21	121.5 (4)
C3—C2—C1	116.6 (4)	O13—N8—O14A	102.2 (8)
C3—C2—H2A	121.7	O13—N8—O13A	44.6 (5)
C1—C2—H2A	121.7	O14A—N8—O13A	114.6 (8)
C2—C3—C4	122.5 (5)	O13—N8—O14	123.2 (6)
C2—C3—H3A	118.7	O14A—N8—O14	42.9 (5)
C4—C3—H3A	118.7	O13A—N8—O14	101.8 (7)
C5—C4—C3	121.5 (4)	O13—N8—C28	119.9 (5)
C5—C4—H4A	119.2	O14A—N8—C28	122.2 (7)
C3—C4—H4A	119.2	O13A—N8—C28	123.2 (6)
C4—C5—C6	116.4 (4)	O14—N8—C28	116.9 (5)
C4—C5—H5A	121.8	O16—N9—O15	122.1 (4)
C6—C5—H5A	121.8	O16—N9—C30	120.3 (4)
N2—C6—C5	131.9 (4)	O15—N9—C30	117.6 (5)
N2—C6—C1	105.5 (4)	O12—C27—C32	125.4 (4)
C5—C6—C1	122.5 (4)	O12—C27—C28	123.1 (4)
N1—C7—N2	112.6 (4)	C32—C27—C28	111.5 (4)
N1—C7—C8	123.0 (4)	C29—C28—N8	116.9 (4)
N2—C7—C8	124.2 (4)	C29—C28—C27	124.6 (5)
O4—C8—C7	105.5 (3)	N8—C28—C27	118.5 (4)
O4—C8—H8A	110.6	C28—C29—C30	119.4 (4)
C7—C8—H8A	110.6	C28—C29—H29A	120.3
O4—C8—H8B	110.6	C30—C29—H29A	120.3
C7—C8—H8B	110.6	C31—C30—C29	121.3 (4)
H8A—C8—H8B	108.8	C31—C30—N9	119.1 (4)
O4—C9—C10	106.1 (3)	C29—C30—N9	119.5 (4)
O4—C9—H9A	110.5	C32—C31—C30	119.3 (4)
C10—C9—H9A	110.5	C32—C31—H31A	120.3
O4—C9—H9B	110.5	C30—C31—H31A	120.3
C10—C9—H9B	110.5	C31—C32—N10	115.8 (4)
H9A—C9—H9B	108.7	C31—C32—C27	123.9 (4)
N3—C10—N4	113.2 (4)	N10—C32—C27	120.3 (4)
N3—C10—C9	123.4 (4)	O17—N10—O18	121.2 (4)
N4—C10—C9	123.3 (4)	O17—N10—C32	119.0 (4)
C16—C11—N4	105.1 (4)	O18—N10—C32	119.8 (4)
C16—C11—C12	123.1 (4)	C33—O19—H19	117.3
N4—C11—C12	131.8 (4)	O19—C33—C34	103.1 (11)
C13—C12—C11	116.2 (4)	O19—C33—H33A	111.1
C13—C12—H12A	121.9	C34—C33—H33A	111.1
C11—C12—H12A	121.9	O19—C33—H33B	111.1
C12—C13—C14	121.4 (4)	C34—C33—H33B	111.1
C12—C13—H13A	119.3	H33A—C33—H33B	109.1
C14—C13—H13A	119.3	C33—C34—H34A	109.5
C15—C14—C13	122.0 (4)	C33—C34—H34B	109.5
C15—C14—H14A	119.0	H34A—C34—H34B	109.5
C13—C14—H14A	119.0	C33—C34—H34C	109.5
C14—C15—C16	117.9 (4)	H34A—C34—H34C	109.5
C14—C15—H15A	121.1	H34B—C34—H34C	109.5
C16—C15—H15A	121.1	C35—O20—H20	103.7
C11—C16—N3	109.6 (4)	C36—C35—O20	116.6 (12)
C11—C16—C15	119.5 (4)	C36—C35—H35A	108.2
N3—C16—C15	130.9 (4)	O20—C35—H35A	108.2
O2—C17—C18	115.4 (6)	C36—C35—H35B	108.2
O2—C17—H17A	108.4	O20—C35—H35B	108.2
C18—C17—H17A	108.4	H35A—C35—H35B	107.3
O2—C17—H17B	108.4	C35—C36—H36A	109.5
C18—C17—H17B	108.4	C35—C36—H36B	109.5
H17A—C17—H17B	107.5	H36A—C36—H36B	109.5
C17—C18—H18A	109.5	C35—C36—H36C	109.5
C17—C18—H18B	109.5	H36A—C36—H36C	109.5
H18A—C18—H18B	109.5	H36B—C36—H36C	109.5

### Hydrogen-bond geometry (Å, °)

**Table:**

*D*—H···*A*	*D*—H	H···*A*	*D*···*A*	*D*—H···*A*
O1—H1W···O5^i^	0.93	1.99	2.758 (4)	139.
O1—H1W···O6^i^	0.93	2.07	2.854 (4)	141.
O1—H2W···O5	0.93	1.99	2.758 (4)	139.
O1—H2W···O11	0.93	2.11	2.898 (4)	141.
O2—H2···O20^ii^	0.95	1.76	2.659 (7)	157.
O3—H3···O17^iii^	0.95	2.09	2.864 (4)	138.
O3—H3···O18^iii^	0.95	2.37	3.289 (5)	163.
N2—H2B···O12^i^	0.88	1.92	2.676 (4)	142.
N2—H2B···O18^i^	0.88	2.25	2.973 (5)	140.
N4—H4B···O15^iv^	0.88	2.10	2.907 (5)	152.
N4—H4B···O16^iv^	0.88	2.38	3.161 (5)	148.
O19—H19···O8	0.93	2.35	3.278 (13)	179.
O20—H20···O19	0.93	1.88	2.807 (13)	179.

Symmetry codes: (i) −*x*+1, −*y*+1, −*z*; (ii) −*x*+1, *y*+1/2, −*z*+1/2; (iii) *x*−1, *y*, *z*; (iv) *x*−1, −*y*+1/2, *z*−1/2.
